# Cross-Cultural Validity of the Child and Adolescent Dispositions Model in a Clinical Sample of Children With Externalizing Behavior Problems

**DOI:** 10.3389/fpsyg.2020.00641

**Published:** 2020-04-08

**Authors:** Teresa Del Giudice, Janina Tervoort, Christopher Hautmann, Daniel Walter, Manfred Döpfner

**Affiliations:** ^1^School of Child and Adolescent Cognitive Behavior Therapy (AKiP), University Hospital Cologne, Cologne, Germany; ^2^Department of Child and Adolescent Psychiatry, Psychosomatics and Psychotherapy, Medical Faculty of the University of Cologne, Cologne, Germany

**Keywords:** child and adolescent dispositions scale, cross-cultural validity, psychometric properties, externalizing behavior problems, children, parent rating

## Abstract

**Background:** The Child and Adolescent Dispositions Scale—parent rating (CADS-P) explores three emotional dispositions that may enlarge the probability of future externalizing problem behavior. The English version has proven its psychometric quality within a population-based sample of children and adolescents. The presents study investigates the German version of the CADS-P by examining a clinically referred sample of children with externalizing behavior problems.

**Methods:**The sample included 132 children aged 4–11 years with a diagnosis of attention- deficit/hyperactivity disorder (ADHD) or oppositional defiant disorder (ODD). The factor structure of the CADS-P was evaluated using exploratory (EFA) and confirmatory factor analyses (CFA). Reliability was estimated using internal consistency (Cronbach's alpha). Validity was assessed through linear regression analyses, with symptoms of externalizing [conduct disorder (CD), ODD, ADHD] and internalizing behavior problems (anxiety, depression) as criterion variables and the three CADS-P factor scores as predictors.

**Results:**After eliminating eight items due to insufficient psychometric properties, EFA and CFA supported a three-factor solution for the German CADS-P. Cronbach's alpha coefficient exceeded α = 0.70 for all subscales. Mostly, as predicted, the CADS-P dimensions were associated with symptoms of ODD/CD and ADHD and symptoms of anxiety and depression.

**Conclusions:**The present study provides evidence for the cross-cultural validity of the CADS- P in a non-English-Speaking country. Results show that the German version of the CADS-P is a reliable and valid parent questionnaire for assessing prosociality, negative emotionality and daring as emotional dispositions that may enlarge the probability to develop externalizing problem behavior.

**Trial Registration:** The study was approved by the review board of the Medical Faculty of the University of Cologne (ID 09-123) and was pre-registered at ClinicalTrials.gov (ID: NCT01350986).

## Introduction

Attention-deficit/hyperactivity disorder (ADHD), oppositional defiant disorder (ODD), and conduct disorder (CD) represent the most common forms of childhood disorders and can be subsumed under the term externalizing behavior problems. According to the Diagnostic and Statistical Manual of Mental Disorders (DSM-5) (American Psychiatric Association, 2013) ADHD is predominantly characterized by inattention and/or hyperactive/impulsive behavior. ODD essentially consists of a persistent pattern of irritability, angry mood, defiant behavior, or vindictiveness, and CD describes a repetitive and persistent pattern of violating the basic rights of others or major age-appropriate societal norms or rules. Epidemiological and longitudinal studies have revealed that ADHD, ODD, and CD often co-occur (Simonoff et al., 1997; Waschbusch, 2002), are highly stable over time and predict numerous negative outcomes in adolescence and adulthood, such as academic underachievement, poor work outcome, criminal activities, substance use, and risky health behaviors (Fergusson et al., 2005; Spira and Fischel, 2005; Biederman et al., 2006; Odgers et al., 2008; Miller and Hinshaw, 2010).

Several risk factors have been identified that may increase the likelihood of externalizing behavior problems. In particular, early temperament factors such as negativity, resistance to control, novelty seeking, and lack of constraint have shown to be predictive for the manifestation of externalizing behavior at later ages (Bates et al., 1991; Krueger and Tackett, 2003; Khan et al., 2005). Regarding the development of CD, Lahey and Waldman proposed the Child and Adolescent Disposition (CAD) model (Lahey and Waldman, 2003, 2005). The model suggests that there are three relatively stable and uncorrelated emotional dispositions that partly explain children's propensity to develop conduct behavior problems: prosociality (i.e., “sympathetic concern for others, helping and sharing, respect for social rules, and guilt over wrongdoings”), negative emotionality (i.e., “easily and intensely upset by frustrations, threats, and losses”), and daring (i.e., “enjoyment of brave, adventurous and risky activities”). According to the CAD model, children who are low in prosociality, high in daring and high in negative emotionality are considered to be at high risk for the development of aggressive behavior problems, especially when confronted with maladaptive environmental influences (Lahey and Waldman, 2003, 2005; Lahey et al., 2008). Furthermore, the development propensity model also formulates hypotheses regarding the comorbidity of CD with other forms of psychopathology, particularly for ODD, depression, and anxiety disorder. It is proposed that these differential forms of psychopathology share similar profiles of emotional disposition with CD. Comparable with CD, children with ODD are also hypothesized to be high in negative emotionality, but to be higher in prosociality and lower in daring (Lahey et al., 2008). Depression and anxiety disorders are assumed to be associated with CD due to their relation to high negative emotionality (Lahey et al., 2008).

To operationalize the three hypothesized socioemotional dispositions and to test the proposed model, Lahey et al. developed and validated the parent-rated Child and Adolescent Dispositions Scale (CADS-P), which consists of 26 items (Lahey et al., 2008). Exploratory factor analyses (EFA) were conducted in a population-based sample of 4–17-year-old children, demonstrating three clear factors (negative emotionality, prosociality, daring) that were consistent with the hypothesized model. Moreover, confirmatory factor analyses (CFA) confirmed the three dispositional dimensions in a second sample. Test-retest reliability of each dimension was high (ICC > 0.80), showing that each disposition can be measured reliably. Additionally, CD symptoms were statistically significantly correlated with negative emotionality, prosociality, and daring. As predicted, the level of CD symptoms was highest in those children who simultaneously showed high scores in negative emotionality and daring and low scores in prosociality. Furthermore, each of the three CADS-P dimensions accounted for unique variance in caretaker-reported ODD symptoms, and prosociality was found to be more strongly associated with CD than with ODD (Lahey et al., 2008, 2010). In addition, as predicted by the model, depression, and anxiety symptoms were both statistically significantly related to negative emotionality (Lahey et al., 2008). In sum, the results support the psychometric quality of the English version of the CADS-P, and the questionnaire thus seems to measure the theoretical constructs for which it was developed. So far, there is no comparable questionnaire in German-speaking countries. Moreover, the association between the dimensions of the CADS-P and symptoms of ADHD has not yet been examined.

Therefore, the first aim of this study was to expand the present knowledge by being the first to investigate its psychometric properties in a non-English-speaking country in terms of cross-cultural validity. A validation of the German version of this instrument would improve our understanding of the factors involved in the development of CD, and may help German mental health professionals to develop better interventions for vulnerable children.

For this purpose, after translation from English into German, EFA and CFA were used to investigate the factor structure of the CADS-P in a German sample of children with externalizing behavior problems. In a first step, the original factor structure of the CADS-P was tested via CFA. In a second step, EFA was used for exploratory purposes in order to determine whether alternative models might fit the data better. To assess reliability, internal consistencies were calculated. The second aim of the study was to examine the association between the CADS-P dimensions and symptoms of a combined ODD and CD score (ODD/CD), ADHD and other psychopathology in terms of external validity. In line with Lahey et al. (2008), we assumed that symptoms of ODD/CD would be positively related to negative emotionality and daring and negatively related to prosociality. For children with ADHD, a high relation with negative emotionality (Healey et al., 2011) and with daring (Lynn et al., 2005) was expected. Depression and anxiety symptoms were assumed to be related to negative emotionality (Lahey et al., 2008).

## Methods

The data analyzed in the current paper were collected as part of a clinical trial that examined the efficacy of telephone-assisted self-help for parents of children with ADHD or ODD (Hautmann et al., 2018). The study was conducted at the Department of Child and Adolescent Psychiatry, Psychosomatics and Psychotherapy at the University Hospital Cologne, Germany, was approved by the review board of the Medical Faculty of the University of Cologne (ID 09-123) and was pre-registered at ClinicalTrials.gov (ID NCT01350986). To increase the sample size, an outpatient sample was additionally recruited at the Department of Child and Adolescent Psychiatry at the University Hospital of Cologne. The following inclusion criteria were applied to both samples: Child (a) aged 4–11 years, (b) attending a kindergarten, primary school, or special school, and (c) meeting the diagnostic criteria for ADHD or ODD according to DSM-IV-TR (American Psychiatric Association, 2000). Exclusion criteria were the child showing (a) an indication or previous diagnosis of borderline intellectual functioning or intellectual disability, (b) an indication or previous diagnosis of a pervasive developmental disorder, and (c) the need for inpatient treatment.

## Study Data

Within the study, data were collected from 101 children (available data at the time of the current analysis). Additionally, data were available from 31 children of the outpatient population. Thus, the total clinical sample comprised 132 children with a mean age of 7.56 years (*SD* = 1.99); 82% were boys. Twenty percent of these children met the DSM-IV criteria for ADHD combined type, 7% for ADHD inattentive type, 21% for ODD, 43% for both ADHD combined type and ODD, and 9% for ADHD inattentive type and ODD. The diagnoses were generated on the basis of a structured interview using the German Diagnostic Checklists for ADHD and ODD, which assess the diagnostic criteria according to DSM-IV and ICD-10 (Döpfner et al., 2008).

## Measures

*The parent-rated Child and Adolescent Dispositions Scale (CADS-P)* is a parent-rated questionnaire which was designed to assess children's emotional disposition toward sympathetic response to others (prosociality), negative emotional response to threat, frustration and loss (negative emotionality), and positive response to novelty and risk (daring). The original version of the CADS-P contains 26 items (see Table 2) that can be aggregated to the three hypothesized dispositions. Prosociality consists of 12 items including concern for others, empathy, and respect for rules. Negative emotionality consists of nine items reflecting mood lability, boredom proneness, and stress reactivity. Daring consists of five items representing risk taking and excitement seeking. The administration of the CADS-P consists of a parent-based report in which the primary caretaker is instructed to rate each of the items on a 4-point Likert- type scale by thinking about how well it describes their child's behaviors and emotions within the preceding 12 months.

For the purpose of this study, the original version of the CADS-P was translated into German. Therefore, the translation was carried out following the guidelines proposed by the International Test Commission (ITC) for the adaptation of test of some cultures to others. Subsequently, the final wording of each one of the items was outlined in order to adapt them to the correct expression in German. Like the original questionnaire, the items were rated on a 4-point Likert scale ranging from 0 (not at all) to 3 (very much). Subscale scores (prosociality, negative emotionality and daring) and a total score were computed by averaging the associated item scores.

*The Symptom Checklist for Oppositional Defiant Disorder and Conduct Disorder—parent rating (Fremdbeurteilungsbogen für Störung des Sozialverhaltens—FBB-SSV)* is part of the German Diagnostic System DISYPS-II, and measures symptoms of ODD and CD according to DSM-IV-TR and ICD-10 (Döpfner et al., 2008; Ise et al., 2014). The parent-report questionnaire comprises 25 items (e.g., “is irritable”) that are rated according to their severity on a 4-point Likert scale ranging from 0 (“not at all”) to 3 (“very much”); higher scores indicate a higher degree of symptoms. Items can be aggregated into two subscales (oppositional behavior and antisocial behavior) and into a total score by averaging the associated item scores. All subscale scores have shown satisfactory internal consistency (0.71–0.93) and factorial validity (Döpfner et al., 2008).

The *Symptom Checklist for Attention-Deficit/Hyperactivity Disorder—parent rating (Fremdbeurteilungsbogen für Aufmerksamkeitsdefizit-/Hyperaktivitätsstörung—FBB-ADHS)* is also part of the DISYPS-II and measures symptoms of ADHD according to DSM-IV and ICD- 10 (Döpfner et al., 2008; Görtz-Dorten et al., 2014). It consists of 20 items (e.g., “has problems in organizing tasks or activities”) that are rated according to their severity on a 4-point Likert scale ranging from 0 (“not at all”) to 3 (“very much”); higher scores indicate a higher degree of symptoms. Items can be aggregated into two subscales (inattention, hyperactivity/impulsivity) and a total score. All subscale scores and the FBB-ADHS total score have shown satisfactory internal consistency (>0.80) and factorial validity (Döpfner et al., 2008; Görtz-Dorten et al., 2014).

The *Child Behavior Checklist* (*CBCL)* (Achenbach and Rescorla, 2001) is a parent-rated questionnaire assessing a broad spectrum of child behavioral and emotional problems. It consists of 118 problem behavior items associated with two superordinate scales: externalizing problems scale (including symptoms of CD, ODD and ADHD; e.g., “can't sit still,” “cruel to animals”) and internalizing problems scale (including anxious and depressed symptoms; e.g., “complains of loneliness”; “too shy or timid”). Each item is rated on a 3-point Likert scale ranging from 0 (“not true”) to 2 (“true”) referring to the child's behavior in the past 6 months. Higher scores indicate a higher degree of symptoms. The German version of the CBCL is a highly reliable rating scale (α = 0.69 to α = 0.93). Further, all subscale scores and the total score have shown factorial validity (Döpfner et al., 2014).

## Data Analyses

The study data were analyzed using IBM SPSS Statistics 23 (SPSS), Mplus (Version 7.4; Muthén and Muthén, 1998–2015 and JASP (Version 0.9.2). Since the range of missing values was low (0.8–10.6%), we did not replace them.

Initially, in the first step, CFA with Mplus was performed to examine the factor structure hypothesized by Lahey et al. (2008). Due to the 4-point Likert scale, item scores were considered as ordered categorical data, and the robust weighted least squares with mean and variance adjustment estimator (WLSMV) was used for model estimation. To handle missing data, the default procedure for WLSMV in Mplus was used (pairwise present analysis). Model fits were evaluated using several fit indices. First, we used the **χ**^**2**^ (chi-square) fit statistic, where a *p* > 0.05 was considered to indicate adequate model fit. Second, we report the comparative fit index (CFI) and the Tucker-Lewis Index (TLI), with a suggested cut-off for acceptable fit of CFI/TLI > 0.90 (Brown, 2006) or respectively, >0.95 (Hu and Bentler, 1999). Third, we calculate the root mean square error of approximation (RMSEA), with a suggested cut-off for acceptable fit of RMSEA <0.08 (Browne and Cudeck, 1992). Additionally, item-to-factor loadings were inspected and were considered to be adequate when standardized factor loadings were >0.35 (Bollen, 1989). In the case of a poor model fit as a result of the CFA, an EFA was performed using SPSS in order to determine whether alternative models might fit the data better. Prior to the EFA, the Kaiser-Meyer-Olkin (KMO) measure was used to assess the adequacy of the sample size and Bartlett's test of sphericity was performed to examine whether the data were suitable for factor analysis. Bartlett's test of sphericity should reach a statistical significance of <0.05 in order to conduct an EFA. The first step of the EFA involved the identification of the number of meaningful factors to retain based on the number of eigenvalues >1 (Kaiser, 1960) and on Cattell (Cattell, 1966). The next step involved a principal components analysis applying a varimax rotation of the retained factors. Low-loading items as well as items that did not correspond to the theoretical construct were then removed and cross-loaded items were retained based on the theoretical grounding of these items and their associated scales. Finally, CFA analyses were reapplied to examine the model fit of the alternative factor structure adapted from the EFA. The reliability of the German CADS-P domain scores was assessed by internal consistency (Cronbach's alpha). According to Moosbrugger and Kelava (2007), the internal consistency of a satisfactory test or scale needs to be at least 0.70. In addition, part-whole corrected item-scale ^correlations^ (*r*_*it*_) were examined with regard to the total scale and the subscales. Values of 0.30 ^≤^ *r*_*it*_^≤^ 0.50 were considered as moderate and values >0.50 as high (Bortz and Döring, 2006). To assess validity, linear regression analyses were conducted. First, the assumptions about residuals (linearity, equality of variance, independence of error and normality) and a residual plot to detect outliers were performed to check that the data were suitable for linear regression analyses. Mean item scores of the oppositional behavior and the antisocial behavior scale (FBB- SSV), the inattention and hyperactivity/impulsivity scale (FBB-ADHS) and the internalizing problems scale (CBCL) were used as criterion variables in separate analyses. In all models, the three CADS factor scores were entered as simultaneous predictors. The proportion of variance accounted for by the three CADS-P dimensions was estimated using *R*^2^.

## Results

### Factor Analyses

The fit statistics for the CFA are presented in Table 1. Model I tested Lahey et al.'s correlated three-factor structure, specifying the dimensions prosociality, daring and negative emotionality (Lahey et al., 2008). It is apparent that the original factor model had very poor fit; the model's RMSEA (0.10) was large and the CFI (0.641) and TLI (0.61) were low. Therefore, an EFA was then performed to determine whether alternative models might better fit the data. A principal component analysis with oblique rotation (promax) was performed on the CADS-P data. The Kaiser-Meyer-Olkin measure verified the sampling adequacy for the analysis, at KMO = 0.75 (Hutcheson and Sofroniou, 1999). According to Bartlett's test of sphericity, the CADS-P items were suitable for factor analysis (*p* < 0.001) (Field, 2009). An initial analysis was run to obtain eigenvalues for each factor in the data. Seven factors had eigenvalues over Kaiser's criterion of 1, but this solution was difficult to interpret. The scree plot showed a point of inflection that would justify three factors. Since a three-factor solution is conceptually consistent with the three subscales, the three-factor solution was further examined. Loadings for the three-factor solution based on the EFA are shown in Table 2. Five of the 26 items (item 3, 12, 14, 18, and 20) had low factor loadings on all factors (<0.35); loading values of the remaining items were above 0.35. The results suggested that factor 1 represented the dimension prosociality, factor 2 the dimension daring, and factor 3 the dimension negative emotionality. In contrast to the findings of Lahey et al. (2008), item 21 showed higher loadings on prosociality than on negative emotionality, item 22 showed higher loadings on daring than on negative emotionality, and item 24 had a salient loading on negative emotionality and not on prosociality. Furthermore, several items showed cross-loadings. According to their loadings and content, the following modifications were made: Low- and non-loading items (item 3, 12, 14, 18, 20) as well as items that did not correspond to the theoretical construct (item 21, 22, 24) were removed and cross-loaded items were retained based on the theoretical grounding of these items and their associated scales. The modified version explained a variance of 53% at a KMO of 0.785. The results of the CFA (see Table 1) indicated a better fit of the modified version of the CADS (model II) compared to the original factor model (model I). According to Browne and Cudeck (1992) the RMSEA value (0.08) is within the acceptable range. Compared to model I, the TLI- (0.88) and the CFI-value (0.90) are markedly higher. However, the TLI-value is below the cut-off of 0.90/0.95 (Hu and Bentler, 1999; Brown, 2006); the CFI value lies in an accepted range according to Brown (2006) but not to Hu and Bentler (1999). Standardized parameters suggested that all items were appropriate indicators of their specific factor (see Table 3). Factors of this model showed only small correlations (*r* = 0.05 to *r* = 0.17), suggesting that the factors represent different constructs and that a higher-order factor was not indicated.

**Table 1:** Confirmatory factor analyses comparing alternative models of the german version of the child and adolescent dispositions scale—parent rating (CADS-P) (Estimator: WLSMV).

**Model**	**χ^**2**^**	***df***	***p***	**TLI**	**CFI**	**RMSEA**
I. Original model (26 items)	1463.205	325	<0.01	0.606	0.641	0.102
II. Modified model (18 items)	1207.855	153	<0.01	0.884	0.900	0.078

*Sample size: n = 132; WLSMV = robust weighted least squares with mean and variance adjustment estimator, χ^2^, empirical χ^2^ value; df, degrees of freedom; p, empirical significance value; TLI, Tucker-Lewis Index; CFI, comparative fit index; RMSEA, root mean square error of approximation*.

**Table 2:** Factor loadings of items on the three factors of the German version of the child and adolescent dispositions scale—parent rating (CADS-P)—extracted in exploratory factor analyses.

		**Factor loadings**		
		**Factor 1**	**Factor 2**	**Factor 3**
**Item**	**Description**			
9	Concerned about others when they are hurt	0.79		
19	Cares about others' feelings	0.79		
17	Feels sorry for kids who get picked on	0.71		
15	Cheers up others	0.64		
5	Would feel guilty if broke laws	0.56		
2	Spontaneously helps others	0.55	0.44	
8	Spontaneously shares	0.54		
10	Emotional	0.51		
11	Would be upset if saw an animal get hurt	0.46		
21	Calm and easy going	−0.44		
18	Wants everyone to follow the rules			
3	Tries to do excellent work			
6	Enjoys risky and dangerous things		0.69	−0.38
7	Likes things that are exciting and loud		0.65	−0.36
4	Likes rough games and sport		0.63	−0.46
1	Daring and adventurous		0.61	−0.50
26	Brave		0.57	−0.47
13	Gets upset easily		0.53	0.46
22	Blow things out of proportion		0.51	
14	Gets bored easily			
25	Moods change unpredictably		0.44	0.57
24	Concerned about right and wrong			0.50
23	Jealous	−0.40	0.37	0.44
16	Reacts intensely		0.42	0.43
12	Easily embarrassed			
20	Enjoys learning interesting things			

*The table displays factor loadings ≥0.35; sorted by height of the loading. Sample size: n = 132*.

**Table 3:** Standardized factor loadings (and standard errors) of the German version of Child and Adolescent Dispositions Scale—parent rating (CADS-P)—Modified model.

	**Prosociality**	**Negative emotionality**	**Daring**
**Item**			
2	0.57 (0.07)		
5	0.45 (0.08)		
8	0.50 (0.07)		
9	0.85 (0.04)		
10	0.57 (0.07)		
11	0.46 (0.08)		
15	0.71 (0.05)		
17	0.73 (0.05)		
19	0.84 (0.04)		
13		0.94 (0.06)	
16		0.75 (0.07)	
25		0.67 (0.06)	
23		0.56 (0.08)	
1			0.71 (0.06)
4			0.80 (0.05)
6			0.84 (0.04)
7			0.74 (0.06)
26			0.68 (0.07)

### Reliability

All three factors of the modified CADS-P model (model II) demonstrated good internal consistencies (see Table 4). Cronbach's alpha (α) and McDonald's omega (ω) exceeded 0.70 for all scales; item-total correlations were mostly moderate to high.

**Table 4:** Descriptive statistics, internal consistencies and part-whole corrected item-scale correlations for the German version of child and adolescent dispositions scale—parent rating (CADS-P)—original and modified model.

**Variable**	**Number of items**	***M***	***SD***	**α**	**ω**	**Range of *r*_**it**_**
Original model (26 items)						
Prosociality	12	1.61^a^	0.44^a^	0.77^a^	0.79^a^	0.15-0.67^a^
Negative emotionality	9	1.78	0.47	0.70	0.72	0.11–0.60
Daring	5	1.61	0.74	0.83	0.83	0.57–0.68
Modified model (18 items)						
prosociality	9	1.83^a^	0.51^a^	0.82^a^	0.83^a^	0.34–0.71^a^
Negative Emotionality	4	1.82	0.66	0.74	0.76	0.41–0.68
Daring	5	1.61	0.74	0.83	0.83	0.57–0.68

*Overall sample size n = 132. M = mean (items rated on a 4-point Likert scale ranging from 0 to 3), SD, standard deviation; α, Cronbach's alpha (internal consistency); ω, McDonald's Omega (internal consistency); r^it^ = part-whole corrected item-scale correlation. ^a^n = 126 (due to missing values).*.

### Validity

Consistent with the CAD model, linear regression analyses revealed that symptoms of oppositional behavior and antisocial behavior (FBB-SSV) were significantly predicted by prosociality (β = −0.22, *t* = −2.35, *p* < 0.05) and negative emotionality (β = 0.74, *t* = 10.64, *p* < 0.01). However, in contrast to the proposed assumption, the symptoms were not significantly predicted by daring (see Table 4).

Symptoms of ADHD (inattention and hyperactivity/impulsivity—FBB/ADHS) were significantly related to negative emotionality, as predicted (inattention: β = 0.20, *t* = 2.13, *p* < 0.05; hyperactivity/impulsivity: β = 0.17, *t* = 2.03, *p* < 0.05). Additionally, symptoms of hyperactivity/impulsivity were significantly related to daring (β = 0.35, *t* = 4.93, *p* < 0.01). As predicted by the model, symptoms of depression and anxiety, measured by the internalizing problems scale of the CBCL, were significantly related to negative emotionality β = −0.27, *t* = 2.19, *p* < 0.05).

The proportion of variance in each dimension of psychopathology accounted for by the three CADS dimensions was estimated using *R*^2^ (see Table 5).

**Table 5:** Test of validity of the three dimensions for the German version of child and adolescent dispositions scale—parent rating (CADS-P)—modified model.

	**FBB-SSV**						**FBB-ADHS**						**CBCL**		
	**Oppositional behavior**			**Antisocial behavior**			**Inattention**			**Hyperactivity/impulsivity**			**Internalizing problems**		
	**β**	***t***	***R*^**2**^**	**β**	***t***	***R*^**2**^**	**β**	***t***	***R*^**2**^**	**β**	***t***	***R*^**2**^**	**β**	***t***	***R*^**2**^**
Parent CADS ratings			0.50			0.23			0.04			0.21			0.02
Prosociality	−0.22	−2.35*		−0.20	−3.05**		0.08	0.61		0.04	0.40		0.11	0.62	
Negative Emotionality	0.74	10.64**		−0.25	5.06**		0.20	2.13*		0.17	2.03*		0.27	2.19*	
Daring	−0.01	−0.13		0.04	0.82		0.11	1.37		0.35	4.93**		−0.02	−0.20	

FBB-SSV, Symptom Checklist for Oppositional Defiant and Conduct Disorder; FBB-ADHS, Symptom Checklists for Attention-Deficit/Hyperactivity Disorder; CBCL, Child Behavior Checklist. *p < 0.05; **p < 0.01.

## Discussion

The present study is the first to examine early developmental factors of CD by investigating the Child and Adolescent Disposition model (Lahey and Waldman, 2003, 2005) within a non-English-speaking country and in a clinical sample of 4–11-year-old children with externalizing behavior disorders. Moreover, to our knowledge, this is the first cross-cultural analysis to investigate the relationship between CADS-P dimensions and ODD/CD, depression/anxiety and ADHD.

The CADS-P was designed to measure three emotional dispositions which have been shown to be associated with a risk of conduct problems in children: prosociality, negative emotionality and daring. Overall, despite differences in the sample composition, our results of the EFA and CFA support a three-factor solution for the German CADS-P, similar to that reported by Lahey et al. (2008). However, in contrast to the findings of Lahey et al. (2008), five items which did not load higher than 0.35 were removed. After these eliminations, all items loaded onto the factors they were intended to measure at moderate to high levels. The results of the CFA indicated better model fit after eliminating these items from the original model. Beyond that, the deletion of items should be viewed with caution given that content validity and scoring facility may be compromised. Here, it is noteworthy that most of these deleted items already showed rather low factor loadings (around *a* = 0.40) in the study by Lahey et al. (2008). Consistent with Lahey et al., the modified CADS-P demonstrated satisfactory internal consistency (Lahey et al., 2008).

The construct validity of the final 18-item version of the German CADS-P was supported by its associations with measures of ODD/CD and ADHD and symptoms of depression and anxiety. In line with Lahey et al. (2008), symptoms of ODD and CD reported in the FBB-SSV were both significantly related to the dimensions prosociality and negative emotionality. Contrary to the model of Lahey et al. (2008), no association was found for the dimension of daring. Notably, Lahey et al. (2008) also found that negative emotionality and prosociality were more strongly related to ODD and CD than daring.

With regard to reported symptoms of ADHD, associations were found with negative emotionality and daring. These results are in line with previous research (Lynn et al., 2005; Healey et al., 2011). Furthermore, as predicted by the model (Lahey et al., 2008), depression and anxiety symptoms (internalizing problems) were related to negative emotionality.

This study has several strengths and limitations. One strength is that despite differences in the sample composition, our study supports the three factor solution of the CADS-P. Moreover, the analysis of a sample of children with externalizing behavior problems, allows the proposed model to be tested in a sample for which the theoretical model was developed. A further strength is that the predictions of the theoretical model were tested using several questionnaires assessing symptoms of ODD/CD, ADHD, and internalizing behavior problems. A limitation is that only parent ratings were used to assess dispositions and symptoms and behavioral and emotional problems. A further limitation of the study is the sample size. Additionally, the composition of the sample differs greatly from that of Lahey's study, which partially makes it difficult to compare the results between the studies.

Overall, this is the first study to provide evidence for the cross-cultural validity of the CADS-P in a non-English-speaking country. Our results show that the German version of the CADS-P is a reliable and valid questionnaire for assessing developmental factors of externalizing problem behavior. Therefore, the use of this instrument during the initial assessment may help to detect factors being associated with externalizing behavior that can inform subsequent treatment plans. Moreover, this instrument could be used during the therapeutic process and may help to identify changes within these factors.

Future studies should try to replicate these finding with large samples of clinically referred children with externalizing behavior integrating a teacher rating. Moreover, a German self-rating version of the CADS-P should be developed as it already exists as an English version.

## Data Availability Statement

'The datasets generated and analyzed during the current study are not publicly available due to obligation to secrecy toward the participants. Queries can be sent to the last author ( manfred.doepfner@uk-koeln.de).

## Ethics Statement

All procedures performed in studies involving human participants were in accordance with the ethical standards of the institutional and/or national research committee and with the 1964 Helsinki declaration and its later amendments or comparable ethical standards. Informed consent was obtained from all children included in the study. Written informed consent was obtained from their parents.

## Author Contributions

TD performed the statistical analysis and drafted the manuscript. JT made substantial contributions to the analysis and interpretation of data. CH and MD designed and coordinated the study. DW was involved in drafting and revising the manuscript. All authors read and approved the final manuscript.

### Conflict of Interest

The authors declare that the research was conducted in the absence of any commercial or financial relationships that could be construed as a potential conflict of interest.
